# The immunology of Parkinson’s disease

**DOI:** 10.1007/s00281-022-00947-3

**Published:** 2022-06-08

**Authors:** Biqing Zhu, Dominic Yin, Hongyu Zhao, Le Zhang

**Affiliations:** 1 Program of Computational Biology and Bioinformatics, Yale University, New Haven, CT USA; 2 Department of Neurology, Yale University School of Medicine, New Haven, CT USA; 3 Department of Molecular, Cellular, and Developmental Biology, Yale University, New Haven, CT USA; 4 Department of Biostatistics, Yale School of Public Health, New Haven, CT USA

## Abstract

Parkinson’s disease (PD) is the second most common neurodegenerative disorder which affects 6.1 million people worldwide. The neuropathological hallmarks include the loss of dopaminergic neurons in the substantia nigra, the presence of Lewy bodies and Lewy neurites caused by α-synuclein aggregation, and neuroinflammation in the brain. The prodromal phase happens years before the onset of PD during which time many patients show gastro-intestinal symptoms. These symptoms are in support of Braak’s theory and model where pathological α‐synuclein propagates from the gut to the brain. Importantly, immune responses play a determinant role in the pathogenesis of Parkinson’s disease. The innate immune responses triggered by microglia can cause neuronal death and disease progression. In addition, T cells infiltrate into the brains of PD patients and become involved in the adaptive immune responses. Interestingly, α‐synuclein is associated with both innate and adaptive immune responses by directly interacting with microglia and T cells. Here, we give a detailed review of the immunobiology of Parkinson’s disease, focusing on the role α-synuclein in the gut-brain axis hypothesis, the innate and adaptive immune responses involved in the disease, and current treatments.

## General introduction and symptoms

Parkinson’s disease (PD) is the second most prevalent neurodegenerative disease throughout the world [1], affecting approximately 1.04 million people in the US [2], and 6.1 million worldwide [3]. The symptoms can be divided into motor features, including bradykinesia, gait disturbance, tremor, rigidity, and speech deficits [4]; and non-motor symptoms, such as depression, hyposmia, cognitive impairment, sleep disorders, and constipation [5]. However, there exists a prodromal phase prior to the onset of PD where people may be asymptomatic or exhibit other symptoms that do not fall into the standard set of PD diagnostic markers [6]. Currently, several prodromal symptoms have been linked to a higher risk of developing PD in an otherwise healthy populations [7]. One of the highest PD risk symptoms is idiopathic rapid eye movement (REM) sleep behavior disorder (RBD), and it has been shown that 80% of the individuals with idiopathic RBD progress to develop PD [6].

PD cases can be classified into two major forms, monogenic and idiopathic. Five to 10% of all cases are monogenic, while the remaining majority are idiopathic [8]. For the monogenic form, 13 loci and 9 genes have been shown to be involved in PD such as Synuclein Alpha (SNCA)/Parkinson Disease 1/4 (PARK1/4) that is associated with sporadic PD and early-onset cases, Leucine-Rich-Repeat Kinase 2 (LRRK2)/PARK8 that has been found in both autosomal dominant PD cases and sporadic cases, Parkin RBR E3 Ubiquitin Protein Ligase (PRKN)/PARK2 which causes early-onset with slow progression, and PTEN-induced kinase 1 (PINK1)/PARK6 which is linked to the autosomal recessive form of PD [9]. In idiopathic PD, there are combination of known environmental factors and genetic elements that consist of many common variants of small effect size across the genome [8]. The pathological hallmarks of idiopathic PD include the death of dopaminergic neurons in the substantia nigra of the midbrain which mainly contribute to motor deficits, and the accumulation of α-synuclein in Lewy bodies and Lewy neurites [10]. Current therapies include dopamine-based treatments (l-DOPA treatment) for motor symptoms and nondopaminergic approaches, like deep brain stimulation, for non-motor symptoms [11]. However, all treatments are only managing symptoms and none of them is curative.

### Epidemiology

The strongest risk factor for PD is aging, with incidence increasing nearly exponentially between ages 55 and 79 [12]. The overall annual incidence rate is around 0.012% for all age groups, while for patients over 50 years of age, the frequency is 0.044% [13]. In addition, the global prevalence is estimated at 0.3% for the overall population but increases dramatically to > 3% for the population of > 80 years of age [14]. However, young onset PD, which refers to disease onset of less than 40 or 50 years of age, is of concern [15]. Around 25% of PD patients experience onset at an age younger than 65 years old, with 5–10% younger than 50 years of age [7].

A large meta-analysis study found that twice as many men than women suffer from PD [16]. Apart from the differences in disease prevalence between the two sexes, men and women show discrepancy in other aspects as well. For instance, other studies showed that women’s age of onset is about 2 years older [16] while men have a steeper increase of incidence as they get older, especially for the age groups of 60–69 and 70–79 [17]. On the other hand, women are more likely to suffer from tremor, dyskinesia, depression, urinary complaints, and constipation [16]. Some factors that may contribute to these differences in disease susceptibility include sex hormone–driven structural differences in the brain, different life-style and environmental risk factors [18], and sex-bias from sex-associated gene mutations [17].

### Pathophysiology

The most well-known and cited hypothesis for PD progression is proposed by Braak’s group [19]. According to this model, PD begins in stages 1 and 2. During stage 1, α-synuclein pathology is found in the olfactory bulb, together with enteric system disfunction and hyposmia. In stage 2, the pathology is present in the medulla, and patients typically show depression. Pathology progresses to the substantia nigra during stage 3, also called a pre-symptomatic phase. REM sleep behavior is considered the main symptom during this phase and afterward, patients begin to manifest motor symptoms. In stage 4, most patients are diagnosed when cortical involvement expands to the temporal mesocortex. Stages 5 and 6 mark advanced PD, which involves the entire cerebral cortex and can lead to impaired cognition and hallucinations in patients.

The pathophysiology of PD results from the complicated interplay of dopaminergic neuron death in the substantia nigra, aberrant intracellular α-synuclein protein aggregations, and neuroinflammation [7]. The loss of dopaminergic neurons causes an imbalance between the indirect pathway over the direct one in the basal ganglia, resulting in pathological synchronous oscillatory activity in the beta band of brainwaves [20]. Recent studies have shown that exaggerated beta oscillation is related to the dopaminergic “off’ state, and may result in the motor symptoms of PD, such as rigidity and bradykinesia [21]. In addition, one hypothesis suggests that a compensatory mechanism involving the lateral premotor loop is caused by the pathological dopaminergic abnormality to account for the brain impairment [22].

Chronic neuroinflammation is one of the salient features of PD pathophysiology, and increased pro-inflammatory factor levels, microglia activation, and T cell infiltration are usually observed in PD autopsy brain sections [23]. Although it may not be the trigger of PD pathology, emerging evidence from human post-mortem PD brains and experimental animal models indicate that α-synuclein aggregations can cause both innate and adaptive immune responses in PD [24]. Consequently, this neuroinflammation can in turn promote α-synuclein misfolding and aggregation [25]. Furthermore, studies suggest that PD neuropathology is also promoted by inflammation in the olfactory system and gut during the prodromal PD phase caused by viral or chemical exposure that can lead to the initial α-synuclein misfolding, aggregation, and propagation to the brain [25]. Besides aggravating the disease, activated microglia can also play an important role in preventing the PD progression. Current immunotherapies targeting α-synuclein rely on clearance and degradation of misfolded α-synuclein deposits by immune cells like microglia [14].

## α-Synuclein and gut-brain axis in PD

Many PD patients show non-motor symptoms related to the gastro-intestinal system, especially during the prodromal phase. To this end, several groups have proposed that the non-motor symptoms may indicate the start of the α-synuclein pathology in the gut, and the α-synuclein will further propagate to the brain via the vagus nerve, causing dopaminergic neuron degeneration and PD.

### Introduction to α-synuclein and the gastrointestinal tract

α-Synuclein is a 140-amino-acid, neuronal protein that concentrates at presynaptic terminals, neuronal nuclei [26], as well as mitochondria, including the inner and outer mitochondrial membranes and the mitochondrial matrix [27]. It can be divided into three distinct domains. First, the *N*-terminal amphipathic region is dominated by four 11-residue repeats including the highly conserved KTKEGV sequence. The central region contains a predominantly hydrophobic motif, called non-amyloid component region, which is indispensable for α-synuclein aggregation [28]. The *C*-terminal region, which is negatively charged and enriched in acid residues [29], is important for chaperone activity and regulation of interaction with other proteins [30]. The function of the α-synuclein protein in normal neurons is controversial and not fully understood. For one, some studies show that α-synuclein expression in neuron terminals has a role in regulation of synaptic plasticity [31]. In tandem, other models suggest that α-synuclein also accumulates in axons after injury and might be associated with regenerative sprouting [32], indicating its function in neuronal remodeling. Yet others observe that α-synuclein acts as a potential chaperone that binds to other proteins to prevent their abnormal aggregation [33]. In addition, it also has a role in mitochondrial function regulation including mitochondrial fusion [34]. Pathologically, α-synuclein is a component of Lewy bodies and Lewy neurites, which can be linked to PD neuropathology both genetically and neuropathologically [31, 35], and it is thought that α-synuclein aggregates into mature Lewy bodies after it forms oligomers and fibrils [35]. Although the exact role of α-synuclein plays in PD remains uncertain, it is likely that the mutation or multiplications of the gene that encodes α-synuclein, namely SNCA, can cause neuronal toxicities that involve disruption and neuronal death, golgi homeostasis, autophagy, or oxidative and nitration stress [35, 36]. Furthermore, a prion-like mechanism hypothesis posits that once misfolded α-synuclein aggregates form in a cell, they can be transferred into other neighboring neurons or brain regions, causing the formation of new aggregations [37], thus contributing to the overall spread of misfolded α-synuclein and disease propagation.

The digestive system mainly comprises the gastrointestinal tract along with other accessory digestive organs [38]. Primarily, the gut wall contains four layers, including the mucosa, submucosa, muscularis externa, and serosa or adventitia [39]. As part of the digestive system, the gastrointestinal tract is involved in food digestion, absorption, and waste excretion [38], as well as immune surveillance through gut-associated lymphoid tissue [40]. Gastro-intestinal function is regulated by hormones such as motilin and ghrelin [41], as well as the autonomic nervous system [39], including the sympathetic and parasympathetic nervous systems. After food uptake, hormones are secreted to facilitate digestion via chemical signaling, and the autonomic nervous system coordinates gastro-intestinal motility, secretions, and intestinal mucosal regeneration [42]. Recently, physiological expression of α-synuclein and phosphorylated α-synuclein have been observed in the gastrointestinal tract, but their properties and functions remain elusive. It has been reported that α-synuclein is mainly expressed in terminals and varicosities of the gut in mouse models where it modulates enteric neurotransmission and development of cholinergic neurons. In contrast, α-synuclein knockout (KO) mice show impaired gastrointestinal functions and elevated enteric neuron density [43]. Other researchers using scanning electron microscopy have shown that α-synuclein can co-localize with synaptophysin in enteric neuron somata and can functionally be linked to the regulation of synaptic vesicle apparatus as well as to synaptic plasticity in enteric neurons [44]. Nevertheless, microbial dysbiosis, or the change in gut microbial composition, has been suggested to be the major source of misfolded α-synuclein in the gut and thus has been connected to the inflammatory processes in PD [45]. Recent research suggests that the bacterial endotoxin, lipopolysaccharide (LPS), may play a critical role in the mediation of the inflammatory process in neurodegenerative diseases such as PD. It has been proposed that genes associated with LPS biosynthesis and type III bacterial secretion systems show significantly more increased expression in PD patients than in healthy controls [46]. Indeed, recent studies demonstrate that compared to mice exposed to LPS-negative α-synuclein fibrils, mice that received intracerebral injection of LPS-positive α-synuclein fibrils produced a specific form of fibrillar α-synuclein, suggesting that synucleinopathies can result from exposure to different pathogens [47].

Gastrointestinal symptoms are common non-motor manifestations in PD patients affecting all regions of the gastro-intestine, including symptoms such as hypersalivation, dyspepsia, constipation, abdominal pain and defecatory dysfunction, with at least one of the symptoms occurring in 60 to 80% of PD patients [48]. These symptoms indicate that PD affects not only the central nervous system (CNS), but also many parts of the peripheral nervous system, such as the enteric nervous system (ENS) and parasympathetic nervous system [49]. Importantly, gastrointestinal symptoms, in particular constipation, is considered one of the earliest and most important symptoms during the prodromal phase, which supports the hypothesis that PD might ate in the gut and then spread to the brain.

### Gut microbial composition in the context of PD

The organisms of the human microbiome have been found in various external and internal parts of the human body, including the gastrointestinal tract, skin, saliva, and other mucosal environments [50]. The majority of these reside in the gastrointestinal tract, with about 10^14^ microorganisms from over 2,000 species [51]. Numerous studies have revealed that the gastrointestinal tract microbiome contributes to immune system development through interactions with the innate and adaptive immune systems [50]. For example, the gastrointestinal tract microbiome has been shown to modulate T helper 17 (Th17) cell differentiation and production. Also, some NOD-like receptors (NLRs) such as NOD-, LRR (leucine‐rich repeat)-, and NOD-like receptor pyrin domain-containing protein 6 (NLRP6) can assemble into inflammasomes in the colonic epithelium to regulate microbiome ecology and intestinal homeostasis [52].

Recently, several studies have shown that compared to healthy controls, PD patients experience metabolic disturbances in the gut, such as alternations in short-chain fatty acids (SCFAs), steroid hormones, and bile acid, which are potentially due to inflammation [53]. For instance, SCFAs modulate gut barrier function, immunomodulation, gut mobility, and obesity [54]. However, along with SCFA-producing bacteria *B. thetaiotaomicron*, SCFAs are found to be reduced in stool from early PD patients, which indicates their potential role in maintaining gut homeostasis [45]. In addition, another metabolite shown to influence dopaminergic neuron functionality is folate [55], which has been discovered to be deficient in late PD patients, causing hyperhomocysteinemia [56].

### Model of gut-brain route

**Fig. 1:**
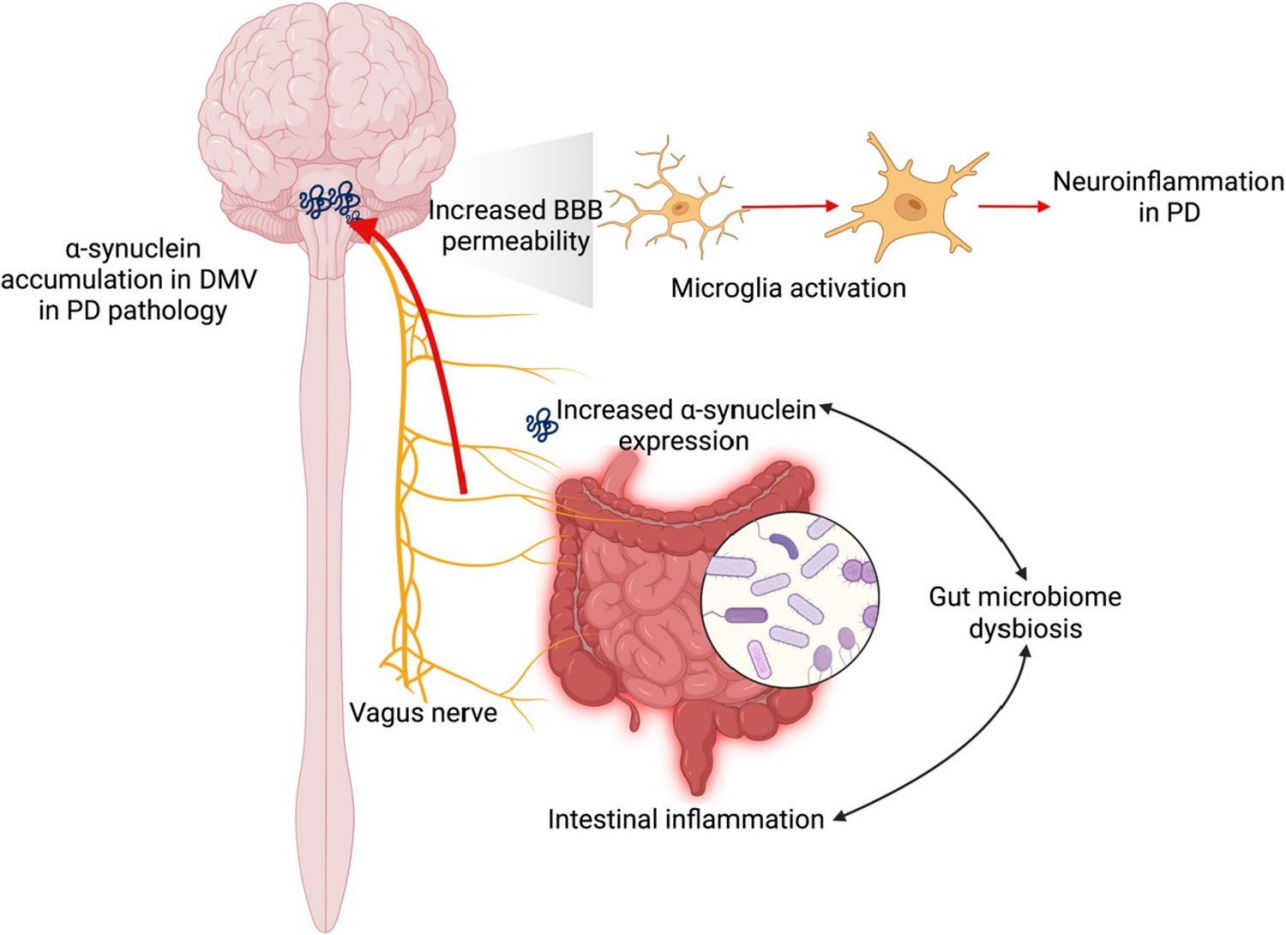
Diagrammed representation of the gut-brain axis hypothesis for the development and progression of PD. An infection or exposure of the gut to toxins can cause preliminary intestinal inflammation and dysbiosis of the gut microbiome. As a result, there is an upregulation of α-synuclein expression and transport through the vagus nerve and into the brain. Increased permeability of the blood–brain barrier (BBB) facilitates the accumulation of α-synuclein within various brain regions, including the dorsal motor nucleus of the vagus nerve (DMV), leading to pro-inflammatory glial responses and the pathogenesis of neuroinflammation during PD

Recently, there is considerable evidence that supports the hypothesis that PD originates in the gut with inflammation and oxidative stress, which then gradually progresses to the CNS [57], which further corroborates Braak’s theory [19]. Indeed, according to the Braak’s hypothesis, PD may be triggered in the gastrointestinal tract by an unknown factor, and then the α-synuclein pathology can propagate through the vagal innervation to the dorsal motor nucleus of the vagus (DMV) and finally the substantia nigra. Here, we describe one possible model of the gut-brain axis in the PD pathogenesis (Fig. 1).

#### An initial inflammatory trigger causes low-level chronic inflammation

Infections caused by toxic substances or other pathogens can trigger the initial inflammation in the intestine. In addition, chronic inflammatory disorders such as inflammatory bowel disease and irritable bowel syndrome have been shown to be linked to an increased risk of PD though the production of proinflammatory cytokines [58, 59]. If the inflammation induced by the triggering factors is not properly reduced, it can lead to dysbiosis of the gut microbiome and increased intestinal permeability, causing the leakage of inflammatory factors from the gut into systemic circulation, and the maturation of antigen-presenting cells. All these shifts can elicit systemic immune responses and increased permeability of the blood brain barrier (BBB) [60].

#### α-Synuclein can transfer from the periphery to the brain and exacerbate inflammation

Inflammation has been shown to have the ability to trigger a progressive increase of α-synuclein expression in the gut [61], and peripheral inflammation enhances α-synuclein uptake from circulation into the brain through altered BBB permeability [62]. Additionally, a recent study demonstrated that α-synuclein can be transported through the vagus nerve to the dorsal motor nucleus after being injected into the rat intestine where the translocation is supported by the microtubule-based axonal transport system [63–65]. In the brain, α-synuclein can trigger microglia activation, which is thought to be one of the most significant signatures of neuroinflammation. Thus, these results indicate that peripheral inflammation accelerates the α-synuclein-induced CNS inflammation.

#### Pathology in PD CNS initially affects DMV

The DMV contains preganglionic cholinergic neurons that innervate the motility of various organs within the gastrointestinal tract [66]. Numerous studies have shown α-synuclein inclusions in the DMV, indicating the spread of α-synuclein from the ENS [67]. According to Braak’s hypothesis, the DMV is involved in stages 1 and 2 of PD development [19]. Later, numerous reports also confirm that in 50–80% of the patients, DMV are involved in the PD pathology as a trigger site [68].

#### PD pathology spreads from one brain region to the next

The inflammation and α-synuclein gradually spread to other parts of the CNS, possibly via a prion-like mechanism, and eventually to the substantia nigra. The spread of synucleinopathy is followed by the loss of dopaminergic neurons in the substantia nigra, and thus the depletion of dopamine [25]. Afterwards, the PD clinical symptoms begin to manifest, from non-motor symptoms to motor dysfunctions.

## The contribution of microglia to PD

**Fig. 2:**
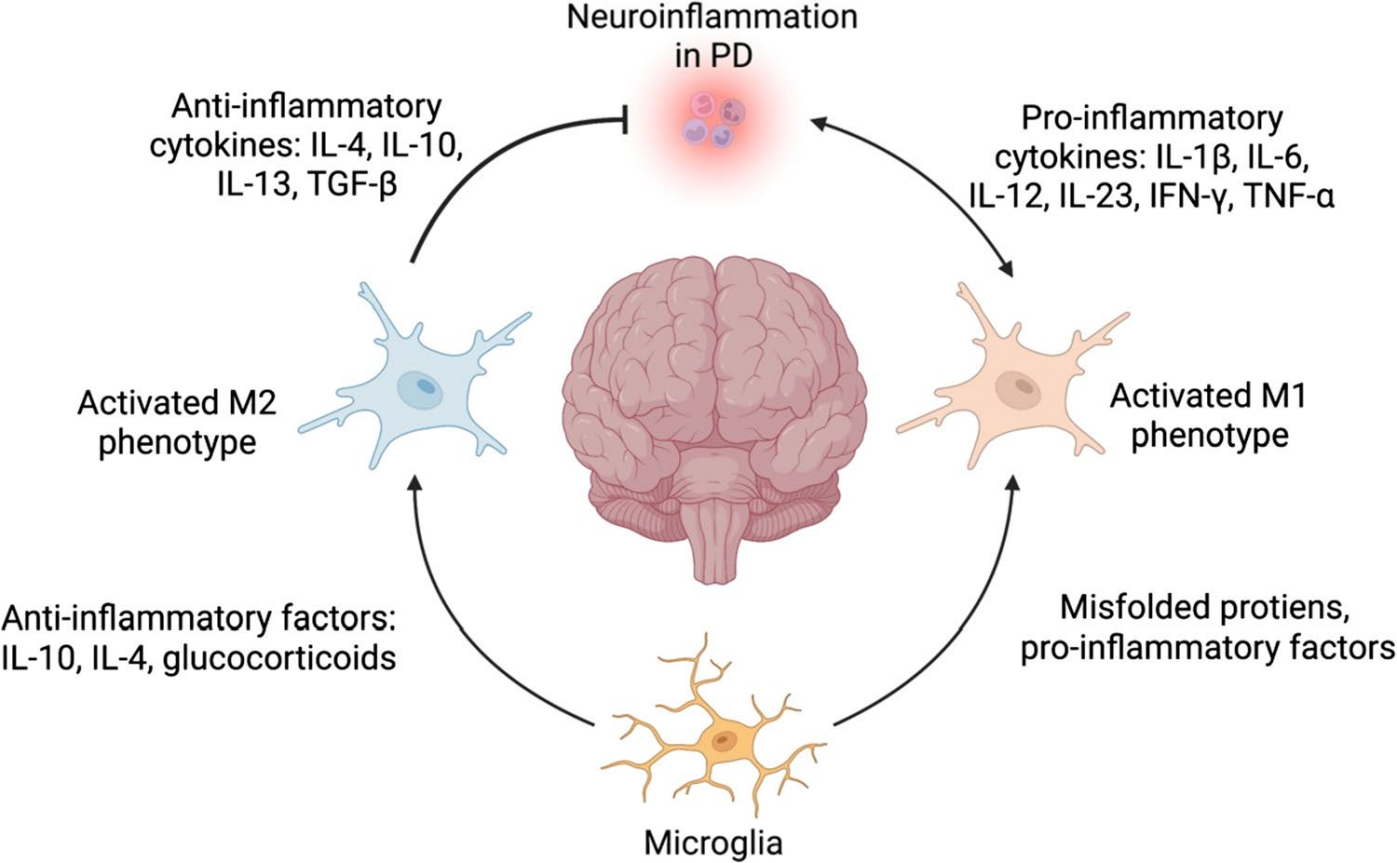
Microglia-mediated neuroinflammation and neuroprotective mechanisms in PD pathogenesis. Microglia become an activated M1 phenotype when exposed to PD pathological conditions like misfolded proteins and pro-inflammatory factors. M1 microglia secrete pro-inflammatory factors that further induces neuroinflammatory and neurotoxic mechanisms in the human brain through process such as enhanced phagocytotic activity and increased ROS production. On the other hand, the presence and stimulation by anti-inflammatory factors can lead to an activated M2 phenotype. Neuroprotective mechanisms in PD from M2 microglia include the release of anti-inflammatory cytokines into the brain which inhibits continued neuroinflammation

Neuroinflammation has been treated as a hallmark of PD and plays a critical role in PD pathogenesis through triggering neuronal dysfunction and death. Specifically, microglia can be activated and further migrate to the brain through a compromised BBB, and contribute to disease progression by mediating the immune pathways and interacting with α-synuclein (Fig. 2).

### Microglia introduction and neuroinflammation

Microglia account for 0.5–16.6% of all the brain cell populations and are the most abundant cell type involved in the immune responses of the CNS [69, 70]. They are small cells that interact with neurons and surrounding cells by exhibiting different functions like synaptic pruning, cerebral angiogenesis, and phagocytosis [71]. Microglia arise from primitive macrophages in the embryonic yolk sac, and enter the CNS through the blood vasculature during embryogenesis [72], constituting an independent cell lineage different from other hematopoetic stem cells. After receiving specific signals from the CNS, the microglia precursor cells start differentiating, maturing, and expressing signature genes [69]. Recent studies have reported that microglia play a crucial role in shaping brain development, especially through their role in eliminating apoptotic cells or neuronal debris through phagocytosis. Since around half of the cells in the brain undergo programmed cell death in the developing CNS, microglia are able to recognize these cells and phagocytose the dead cell corpses [73]. Another important role of microglia is developmental synapse pruning, which results from the competition between neighboring neurons to eliminate excess synapses and maintain more active synaptic connections [74]. To this end, researchers have shown that impaired synaptic pruning can cause an increase in spine density and immature neural circuits [75]. Furthermore, numerous single-cell RNA sequencing studies have recently revealed the diversity of microglial cellular heterogeneity, such as differences in regional density and function [76]. Studies have demonstrated that microglial density varies across brain regions, with more microglia in gray matter compared to white matter. In addition, the hippocampus, olfactory bulb, basal ganglia, and substantia nigra have denser microglia as compared to the fiber tracts, cerebellum, and brainstem. Though the reason why microglia density varies across brain regions is still not clear, some hypothesize that it may relate to microenvironmental regulation or the glia-to-neuron ratio [76, 77].

Similarly, microglial function varies across CNS regions as well. For example, several studies suggest that microglia show different functional profiles between gray matter and white matter, with higher expression of genes involved in type-I interferon response in gray matter microglia and higher level of genes belonging to the nuclear factor kappa B (NF-κB) pathway in white matter microglia [78]. Taken together, the diverse functional heterogeneity of microglia may contribute to the reaction towards location-dependent pathological stimuli in the brain [79]. Reactive microglia were first found in the substantia nigra of human post-mortem PD brains in 1988 [80]. Subsequently, other studies confirmed the presence of reactive microglia in the substantia nigra of PD patients. Positron emission tomography studies also found widespread, activated microglia in different brain regions, such as the brainstem, basal ganglia, and frontal areas [81]. Furthermore, microgliosis in substantia nigra and striatum [23] has been observed in several PD mouse models. Reactive microglia in different brain regions accompanied by higher levels of pro-inflammatory cytokines, such as tumor necrosis factor-α (TNF-α), interleukin-1β (IL-1β), interleukin-6 (IL-6), and interferon-gamma (IFN-γ), suggest the involvement of microglia in neuroinflammation during the development of PD [71]. Upon activation or microgliosis, microglia exhibit two diametric phenotypes, either the M1 pro-inflammatory phenotype or the M2 anti-inflammatory phenotype. Pro-inflammatory M1 microglia can be induced by misfolded proteins and environmental toxins, and are characterized by the functions of phagocytosis, apoptotic cell debris removal, as well as production of pro-inflammatory cytokines such as IL-1β, TNF-α, interleukin-12 (IL-12), and interleukin-23 (IL-23) [82]. In contrast, immunosuppressive M2 microglia produce anti‐inflammatory cytokines, for example, interleukin-10 (IL-10), interleukin-4 (IL-4), interleukin-13 (IL-13), and transforming growth factor-β (TGF-β), the roles of which are to suppress inflammation, restore homeostasis, and promote repair [23]. In fact, neuroinflammation is thought to be a “double-edged sword.” Activation of microglia is protective and helps to remove foreign pathogens and toxins. Inversely, chronic microgliosis can contribute to cytotoxicity and neuronal loss in PD [83]. In fact, long-term, over-activation of microglia accelerates cellular stress, affects memory, and impairs neuronal plasticity. And with the progression of PD, apoptotic neuronal cells release matrix metalloproteinase-3 (MMP-3), ATP, and α-synuclein, which in turn further activates microglia, resulting in PD neuron degeneration [84]. For example, ATP released by injured neurons and surrounding astrocytes binds to the P2Y receptor in microglia, and regulates the chemotaxis of microglia towards injury [85].

### Activation of microglia by α-synuclein

Multiple studies have shown that extracellular α-synuclein positively regulates microglia activation as well as inflammatory responses, and microglia are able to phagocytose α-synuclein for degradation [86]. During PD pathology, over-produced or misfolded α-synuclein is secreted in many regions of the brain by neurons which activates microglia, leading to pro-inflammatory responses with elevated levels of cytokines including IL-1β, IL-6, and TNF-α, the production of free radicals, and contributions to neuron toxicity [87], which subsequently drives the PD progression. Research has also shown that microglia activation and cytokine (TNF-α and IL-1β) production rely on the fibrillar form of α-synuclein, compared with oligomeric and monomeric forms [88].

Several reports have suggested that some immune pathways in microglia are related to α-synuclein activation. For example, phosphate oxidase (PHOX), a reactive oxygen species (ROS)–generating enzyme, can be activated in microglia by α-synuclein, which induces strong ROS production and contributes to elevated neurotoxicity [87]. Another example is Galectin-3, a member of the galectin lectin family, which plays an important role in inflammation regulation. Galectin-3 contributes to α-synuclein-induced activation of microglia, with downregulation causing significant inhibition of microglia activation [89]. Additionally, α-synuclein evokes the nucleotide-binding oligomerization domain (NOD)–like receptor pyrin domain-containing protein 3 (NLRP3) inflammasome in microglia through dual stimulation [90], and further enhances IL-1β secretion.

However, studies reveal that different forms of α-synuclein cause various degrees of microglial phagocytosis and microgliosis intensity [71]. For instance, A30P or A53T mutant α-synucleins trigger a stronger microglia immune response than wild-type α-synuclein as demonstrated by increased microglial secretion of TNF-α, IL-6, but compromises phagocytosis [87]. In particular, A53T mutant rapidly induces microgliosis through the recruitment of mitogen-activated protein kinase (MAPKs), NF-κB, activator protein 1 (AP-1) and subsequently the activation of nuclear factor erythroid 2–related factor 2 (Nrf2). In contrast, physiological monomeric α-synuclein boosts phagocytosis [91] and promotes anti-inflammatory microglial functions through decreasing extracellular signal–regulated kinase (ERK) activation and increasing peroxisome proliferator-activated receptor γ (PPARγ) pathway activity.

### Microglia as a phagocytic cell clearing α-synuclein

Microglia phagocytose neuronal debris, pathogens, and unfolded proteins in the brain, which is crucial for neural development and homeostasis [92]. Phagocytosis is a receptor-mediated process, during which receptors first recognize phagocytic targets, the target is then engulfed, and finally the particle-enclosed phagosome is digested through fusion with lysosomes [93]. Indeed, several receptors have been proven to mediate microglial phagocytosis. For example, the toll-like receptors (TLRs), such as TLR4, are important in controlling α-synuclein uptake, and ablation of TLR4 promotes α-synuclein overexpression [94]. On the other hand, anti-TLR2 antibody has been shown to increase microglia phagocytosis in an Alzheimer’s disease (AD) model [95]. In addition, triggering receptors expressed on myeloid cells 2 (TREM-2) signals the phagocytosis of both apoptotic neurons and other cell debris [71].

Previous studies have suggested that microglia is the main cell type clearing α-synuclein to mitigate the spread of the aggregates to neighboring cells [96]. A list of proteins has been suggested to be responsible for the microglia phagocytosis of α-synuclein. In fact, microglia internalize extracellular monomeric α-synuclein via the monosialotetrahexosylganglioside (GM-1) receptor and other receptors [97], whereas internalization of aggregated α-synuclein involves the coated vesicle formation protein clathrin. However, the degradation of fibrillar α-synuclein is shown to be slower than α-synuclein engulfment, leading to the accumulation of aggregates within cells [98], and protein deposition.

### Cascades initiated by α-synuclein in microglia

#### α-Synuclein/TLRs/NF-κB/NLRP3 axis

The signaling cascade of microglia activation initiated by α-synuclein is complicated, involving TLRs, NF-κB, NLRP3, and possibly other cascades [99]. TLRs are one class of pattern-recognition receptors (PRRs) which recognize pathogen-associated molecular patterns (PAMPs) in addition to damage-associated molecule patterns (DAMPs) [100] like that of α-synuclein. Moreover, NLRP3 inflammasomes are a group of protein complexes that can induce neuroinflammation and cell death [101]. Upon α-synuclein recognition by TLR2 or TLR4 in microglia, a phosphorylation cascade is initiated which leads to the translocation of NF-κB [102], and the activation of the NLRP3 inflammasome, which itself causes microglia activation.

#### Nrf2

Recent study suggests that Nrf2-directed antioxidant response system plays an important role in PD in response to α-synuclein. Nrf2 is an antioxidant transcription factor that plays an important role in cellular antioxidant response by regulating detoxification and antioxidant enzymes [103]. Accordingly, monomeric A53T α-synuclein–induced microgliosis is regulated by phosphorylation mechanisms where MAPKs, NF-κB, AP-1, and Nrf2 are engaged [104]. Additionally, in the Nrf2 impaired mouse model, microglia showed increased pro-inflammatory markers but failed to activate the two antioxidant enzymes, heme oxygenase-1 (HO-1) and nicotinamide adenine dinucleotide phosphate quinone oxidoreductase-1 (NQO1) [105].

#### Major histocompatibility complex class II (MHC class II)

MHC class II binds antigenic peptides that are processed in endosomes and present them on the cell surface for CD4 T cell recognition [106]. In the brain, activated microglia can act as the main antigen presenting cells [107]. In this regard, α-synuclein triggers significantly increased expression of MHC class II in microglia, followed by enhanced antigen processing, and finally CD4 T cell proliferation [108]. Conversely, MHC class II knock-outs show reduced microglia activation in response to α-synuclein and neurodegeneration [108].

## T cell contribution to PD

T cells were found in the substantia nigra of PD patients more than a decade ago [109], and have been shown to shape the pathogenesis of PD through their involvement in the adaptive immune responses in the gut and brain. In particular, different mediators can induce T cell-driven inflammation, such as α-synuclein, gut microbiota, dopamine, and SCFA, and the inflammation eventually progresses to the brain.

### T cell recognition of α-synuclein

#### Two antigenic regions in α-synuclein

Recent studies have shown that T cells recognize epitopes derived from α-synuclein, suggesting the adaptive immune response plays a role in PD pathogenesis [110]. Specifically, two antigenic regions were identified in α-synuclein. The first is near the *N*-terminus and is known as the Y39 region, which includes two epitopes, and the second is the S129 region, near the *C*-terminal and is composed of three epitopes. The T cell response to α-synuclein antigenic peptides is largely mediated by IL-5 or IFNγ-secreting CD4 T cells, as well as IFNγ-secreting CD8 cytotoxic T cells.

Approximately 40% of the PD patients in the cohort exhibited immune responses to α-synuclein epitopes, which may reflect variations in disease progression or environmental factors. T cells can be activated by the α-synuclein epitopes from both the extracellular native α-synuclein presenting in the normal blood, and the fibrilized α-synuclein associated with PD.

#### HLA alleles that present α-synuclein peptides

Using an in vitro binding assay, HLA alleles that present α-synuclein have been identified, including HLA class II variants DRB1*15:01 and DRB5*01:01, which are in linkage disequilibrium. In general, PD patients showed a higher expression of HLA molecules, particularly HLA class II, in agreement with the findings that the HLA class II may enhance the PD susceptibility by inducing a more inflammatory environment [111]. Genome-wide association studies also show the association of PD with the immune haplotype of HLA class II variants DRB1*15:01 and DRB5*01:01 [112], which can bind the α-synuclein Y39 region with high affinity and the S129 region with low affinity. On the other hand, the HLA class I allele A*11:01, in mild linkage disequilibrium with the two HLA class II variants, can be bound by a shorter α-synuclein peptide in the Y39 region with high affinity. Thus, immune responses of PD patients to α-synuclein have both MHC class I and II components.

#### T cell reactivity to α-synuclein is linked to preclinical and early motor PD

From a longitudinal case study, it has been shown that elevated α-synuclein-specific T cell responses were detected prior to the diagnosis of motor PD and then waned [113]. During this study, the peripheral blood mononuclear cell samples of a single individual collected multiple times before and after the diagnosis of motor PD were analyzed. Surprisingly, a strong CD4 T cell reaction against α-synuclein epitopes was detected more than 10 years before the PD diagnosis, whereas in the samples after diagnosis, the T cell reactivity was significantly lower. In a further study, two additional PD patient cohorts were examined, and it was found that T cell responses to α-synuclein were strongest shortly after PD diagnosis and produced high level of cytokines (IFN-γ, IL-5, and IL-10), and the reactivity declined afterwards. Overall, these studies suggest that α-synuclein-specific T cells in PD are most abundant immediately after diagnosis of motor PD.

#### The T cell receptor (TCR) repertoire of α-synuclein-specific T cells

To further characterize α-synuclein-specific TCR clonotypes, the TCR repertoire from PD patients were mapped and compared. There were no defined, shared clonotypes among patients which indicated α-synuclein-specific TCR repertoire may be diverse and patient-specific [114]. Immunomodulatory interventions can be used to modify specific T cell responses; thus, future studies to match PD specific HLA alleles with antigen-specific TCRs may provide novel immunotherapies and diagnostic tools for treating PD and tracking the disease progression.

### Factors regulate T cell-mediated immunity in PD

#### Gut microbiota

It has been shown that some particular components of gut microbiota can bypass TLRs and directly induce Th17 differentiation through adhesion to intestinal epithelial cells [115]. Moreover, they can facilitate the generation of Tregs and thus mediate the balance between pro- and anti-inflammatory activities [116]. In another study, the researchers found that germ-free mice develop neuroinflammation and physical impairments when treated with microbiota from PD patients [117]. Interestingly, mouse models show that the administration of the bacterium, *Proteus mirabilis*, from PD mice can cause dopaminergic neuron damage, neuroinflammation, and α-synuclein aggregation [118], and the excessive α-synuclein can further cause T cell activation as stated in the previous section.

#### Dopamine

Dopamine is a neurotransmitter and neuromodulator that controls brain functions including reward, hormone secretion, and movement regulation [119]. It triggers cell function through dopaminergic receptors (DRs), and gut microbiota is one of the major dopamine sources in the gut [120]. CD4 T cells have been shown to possess all five types of DRs: DR1–DR5, with different DR stimulation causing different T cell activation and cytokine synthesis [121]. PD animal models show that DR3-deficiency in CD4 T cells protect mice from 1-methyl-4-phenyl-1,2,3,6-tetrahydropyridine (MPTP)-induced neurodegeneration [122], while high levels of dopamine triggers the secretion of anti-inflammatory cytokine IL-10 in CD4 T cells through stimulating DR2 [123].

#### SCFA

SCFAs, including acetate, propionate, and butyrate, can regulate T cell activation through G-protein-coupled receptors (GPCRs) or histone deacetylase (HDAC) inhibition [124], allowing them to exert an anti-inflammatory effect in the gut. For example, SCFA can impair Th2 responses through GPR41, and therefore alter immune responses [125]. Another study shows that colonic Tregs can be mediated by SCFA through GPR43 and protect against colitis in mice [126].Regarding HDAC inhibition, evidence suggests that butyrate and propionate can lead to Foxp3 acetylation and further increased Treg differentiation [116]. More recently, some have hypothesized that the dysbiosis and SCFA alternation in PD patients are related to intestinal inflammation. Indeed, deficiencies in SCFAs are found in PD fecal samples compared with healthy controls [127] which may lead to impaired Treg activities and PD pathogenesis.

### T cell infiltration into the brain

T cells can migrate to the CNS through the BBB and induce immune responses in many autoimmune diseases and CNS infections. Certain chemokines are crucial for T cell brain infiltration. For instance, C-X-C motif chemokine receptor 3 (CXCR3) plays an important role in T cell recruitment to the CNS by binding with C-X-C Motif Chemokine Ligand 10 (CXCL10), CXCL9, and CXCL11 in experimental autoimmune encephalomyelitis (EAE) [128]. Apart from chemokines, integrins also have a key role in mediating T cell adhesion during T cell migration through the BBB. Specifically, α4β1-integrin on Th1 and Th17 cells binds with endothelial vascular cell adhesion molecule 1 (VCAM-1) for transmigration across the BBB [129]. Other groups have shown that αvβ3 and αLβ2 integrins may also be responsible for the migration [130].

**Fig. 3:**
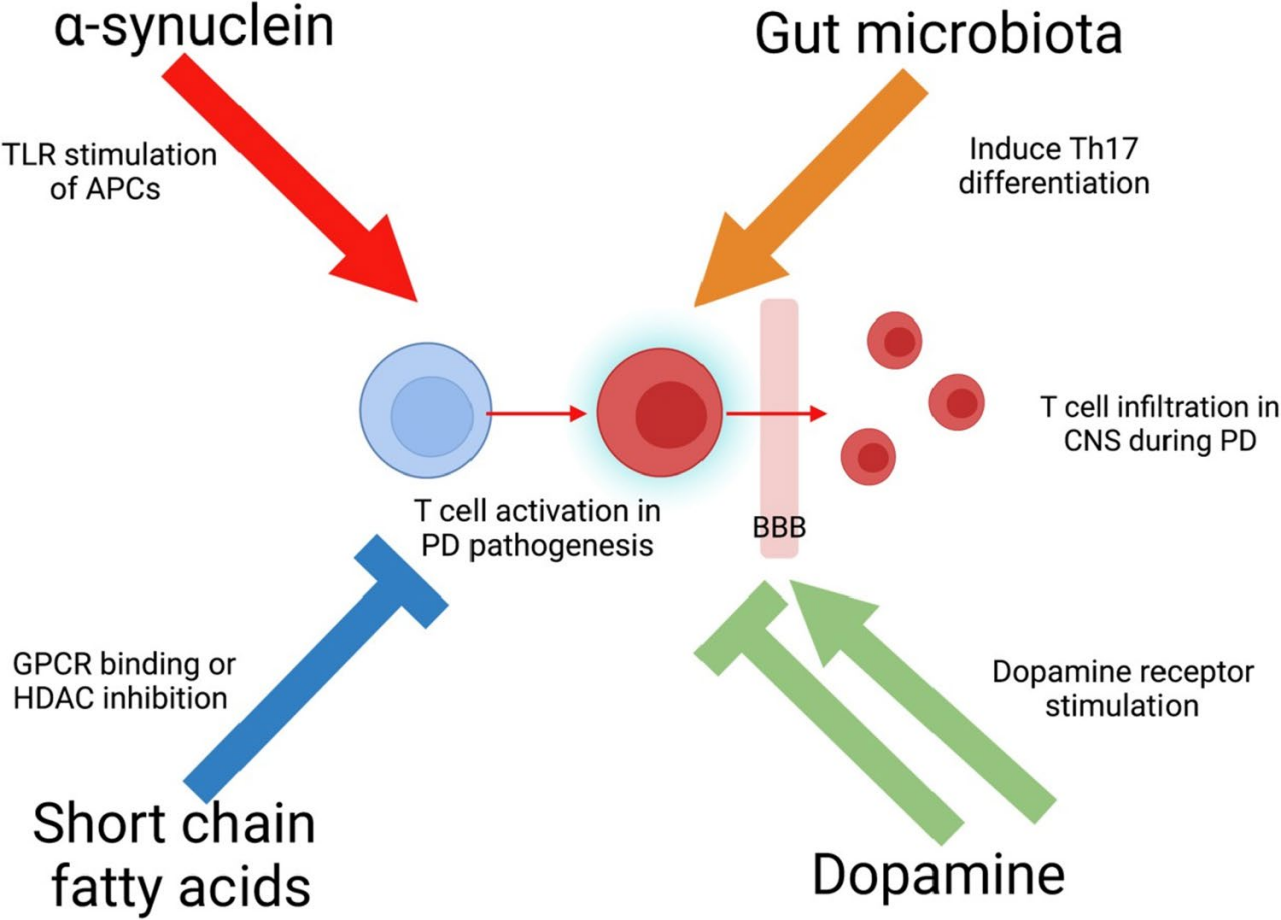
Inflammatory factors that regulate T cell–mediated immunity in PD progression. Primarily originating from the gut, various mechanisms such as α-synuclein, gut microbiota, dopamine, and short-chain fatty acids (SCFAs) can result in the activation of T cells that can then bypass a leaky blood–brain barrier (BBB) and travel into the brain. Infiltrated T cells induce neuroinflammation through the secretion of pro-inflammatory cytokines and activation of microglia, leading to the pathogenesis of neurodegenerative diseases like PD

There are increasing reports of T cell infiltration in neurodegenerative diseases such as PD andassociate this infiltration with dopaminergic neuron degeneration [131]. In fact, several studies have verified the connection between CD4 T cell infiltration and dopaminergic cell loss in mouse models. For example, mutant mice that lack T and B cells are both resistant to neuronal loss [132]. Neuroinflammation, as well as neurodegeneration, can be attenuated by the transfer of Tregs from copolymer-1-immunized mice, which suppresses reactive microglial responses [133]. Moreover, a recent study concluded that infiltrating CD8 T cells are increased in PD brain, and some of these cells contact dopaminergic neurons and cause neuronal death [134] (Fig. 3).

Cerebrospinal fluid (CSF) is contained in the brain ventricles and spinal cord, and considered to be a fluid envelope that protects the central nervous system [135, 136]. CSF is predominantly composed of T cells, which provide critical immune surveillance of the central nervous system, and also contains other immune cells, such as B cells and myeloid cells [137]. Although elevated levels of T cells has been found in PD mouse models [109] and patient postmortem brain samples [138], the T cell composition and the exact role of these T cells are still being investigated. It has been shown that activated T cells were increased in the CSF of PD patients compared to healthy controls, along with the enhanced levels of pro-inflammatory cytokines including interleukin-2 (IL-2), IL-6, and TNF-α, demonstrating the involvement of adaptive immune response in PD development [135]. Furthermore, another study focusing on TCRs in the CSF discovered clonal expansion of T cells in PD compared to controls, especially for CD8 T cells [139], similar to what was reported in the CSF of AD patients [140]. Thus, these results provide evidence for the importance of T cell surveillance in the CSF of PD patients, and highlight the need to further understand the interactions between the adaptive immune system and the central nervous system.

## PD treatments

### Treatment targeting inflammatory pathways

Drugs have been developed to target the inflammatory pathways mediated by activated immune cells. Previous results show that the anti-inflammatory drug dexamethasone prevents glial cell activation and exerts a protective effect against dopaminergic degenerative processes [141]. Similarly, naloxone inhibits microglia activation and pro-inflammatory cytokine production to protect dopaminergic neurons as well as other neurons [142]. In addition, non-steroidal anti-inflammatory drugs (NSAIDS) also have protective effects against neuronal damage. For example, aspirin prevents dopaminergic depletion and neuronal damage by inhibiting ROS production. Celecoxib inhibits microglial activation through the inhibition of cyclooxygenase (COX-2) in order to protect dopaminergic neurons from degeneration [143].

### α-Synuclein-related treatments

Two important treatments related to α-synuclein either increase α-synuclein clearance or offer neuroprotection by α-synuclein vaccination. Monophosphoryl lipid A (MPLA), a TLR4-selective agonist, has been shown to induce increased α-synuclein uptake by microglia through TLR4, and thus can reduce α-synuclein aggregation as well as rescue dopaminergic neurons [144], thereby increasing α-synuclein clearance. As for α-synuclein vaccination, α-synuclein/glucose-related protein 94 (Grp94) combination vaccination has the ability to reshape the disease immune environment by suppressing microglial activation and neuroinflammation in a PD mouse model [145].

## Conclusions

Parkinson’s disease is the second most common neurodegenerative disease characterized by the progressive loss of dopamine neurons in the substantia nigra along with the aggregation of intraneuronal Lewy bodies and neurites, leading to motor and non-motor symptoms. α-Synuclein plays an important role in initiation and progression of PD, and may also be involved in the gut-brain route model. Both innate and adaptive immune responses are triggered during the PD pathogenesis, with the hallmarks being microglia and T cell activation. Finally, new treatments of PD are continually being developed and the most well-known therapies can treat motor or non-motor symptoms, as well as target key inflammatory pathways and modulate α-synuclein.
